# Sparse conserved under-methylated CpGs are associated with high-order chromatin structure

**DOI:** 10.1186/s13059-017-1296-x

**Published:** 2017-08-31

**Authors:** Xueqiu Lin, Jianzhong Su, Kaifu Chen, Benjamin Rodriguez, Wei Li

**Affiliations:** 1 Division of Biostatistics, Dan L Duncan Cancer Center, Baylor College of Medicine, Houston, TX 77030 USA; 2 Department of Molecular and Cellular Biology, Baylor College of Medicine, Houston, TX 77030 USA; 3 Department of Bioinformatics, School of Life sciences and Technology, Tongji University, Shanghai, 20092 China

**Keywords:** Sparse conserved under-methylated CpG, Whole-genome bisulfite sequencing, Multi-sample-based method, Chromatin-loop factors, Interacting anchor, DNA methylation, Chromatin structure

## Abstract

**Background:**

Whole-genome bisulfite sequencing (WGBS) is the gold standard for studying landscape DNA methylation. Current computational methods for WGBS are mainly designed for gene regulatory regions with multiple under-methylated CpGs (UMCs), such as promoters and enhancers.

**Results:**

To reliably predict the functional importance of single isolated UMCs across the genome, which is usually not achievable using traditional methods, we develop a multi-sample-based method. We identified 9421 sparse conserved under-methylated CpGs (scUMCs) from 31 high-quality methylomes, which are enriched in distal interacting anchor regions co-occupied by multiple chromatin-loop factors and are flanked by highly methylated CpGs. Moreover, cell lineage-specific scUMCs are associated with essential developmental genes, regulators of cell differentiation, and chromatin remodeling enzymes. Dynamic methylation levels of scUMCs correlate with the intensity of chromatin interactions and binding of looping factors as well as patterns of gene expression.

**Conclusions:**

We introduce an innovative computational method for the identification of scUMCs, which are novel epigenetic features associated with high-order chromatin structure, opening new directions in the study of the inter-relationships between DNA methylation and chromatin structure.

**Electronic supplementary material:**

The online version of this article (doi:10.1186/s13059-017-1296-x) contains supplementary material, which is available to authorized users.

## Background

DNA methylation is an important epigenetic modification involved in both normal development and disease. In the whole genome, highly methylated CpG dinucleotides govern most of the methylome (70–80%) [1, 2], except in gene regulatory regions which are characterized by intermediate to low levels of methylation. Single-base methylome analysis by whole-genome bisulfite sequencing (WGBS) has led to the recent proposal of several epigenotypes that characterize the nature and function of DNA sequences with reduced cytosine methylation, including low methylated regions (LMRs) [3], hypo-methylated regions (HMRs) [4], under-methylated regions (UMRs) [4], and DNA methylation canyons [5] and valleys (DMVs) [6]. These hypomethylated regions are often both enriched in transcriptional regulatory elements such as promoters, enhancers, and transcription factor binding sites (TFBSs) as well as subject to aberrant changes in tumorigenesis. A defining property common to the last three epigenotypes is the extent of low methylation (mCG ≤ 10%). Thus, they may be broadly considered classes of UMR. Given that methylation of a single cytosine position in supercoiled DNA has been shown to be sufficient to alter the kinetics of local DNA helix stability in vitro [7], it surprised us that current computational methods cannot reliably predict genome-wide very short, functional UMRs containing less than four under-methylated CpGs (UMCs) [2, 5]. For example, they can either be ignored by algorithmic design [5] or discarded following correction for multiple hypothesis testing [2] (Additional file 1: Figure S1a).

Additional strategies for identifying gene transcriptional regulatory regions also include Dnase I Hypersensitive sites (DHSs) [8] as well as chromatin modifications assayed by chromatin immunoprecipitation sequencing (ChIP-seq) [9, 10]. The majority of DHSs are associated with local hypomethylation. If we consider the frequency of co-occurrence between a given set of UMRs (predicted without false discovery rate [FDR] correction) and DHSs in a particular cell model, the proportion of short, single-base UMRs found in DHSs is low relative to that of larger UMR (Additional file 1: Figure S1b). However, the absolute number of these single-base UMRs is approximately threefold greater than that of larger UMRs (Additional file 1: Figure S1c) [2], suggesting there is a considerable number of functional sequence elements with regulatory potential subject to epigenetic control that have been missed by previous methylation studies. Among these methods for investigating regulatory regions genome-wide, WGBS is unique in that it provides information at single-base resolution. We can map single-base UMCs, but no published methods can predict their functionality. Hidden Markov Model (HMM) [3–5] and window-based [6] methods are frequently used to identify regions of low methylation. But these two methods will not be effective because they are agglomerative and depend on the correlation of methylation levels between adjacent CpGs [11]. Simply put, functional single-base UMCs, by definition, do not cluster together as the UMCs in larger UMRs.

The genome possesses three-dimensional (3D) organization in nuclear space, which regulates transcription by facilitating interactions between gene promoters and distal regulatory elements within large topological domains [12–17]. Chromatin loops are used to describe the long-range interactions within topological domains that connect distal regulatory elements with target promoters [9, 18]. Cohesin protein complex (RAD21 and SMC3), CTCF, and ZNF143 are four major factors involved in the establishment and maintenance of long-range interactions. In fact, most of the anchors of chromatin loops mapped in human cells are co-bound by these four factors together [14, 19] (Additional file 2: Table S1). The mechanisms through which these four factors mediate high-order chromatin structures are partially understood (Additional file 2: Table S2). Several studies have shown that the deletion or inversion of CTCF sites is enough to disrupt the corresponding chromatin loop and alter gene expression [20–22]. Furthermore, some proto-oncogenes (such as *PDGFRA* [23], *TAL1*, and *LMO2* [24]) can be activated by the deletion of CTCF sites at the boundaries of topological domains. ZNF143, a more recently characterized chromatin-loop associated factor, provides sequence specificity to secure chromatin interactions at gene promoters, interactions which are disrupted by single-nucleotide polymorphisms (SNPs) at ZNF143 motif sites [25]. Notably, the binding of chromatin-loop factor CTCF is methylation-sensitive [26]. Two recent studies focusing on specific gene loci demonstrated that DNA methylation of CTCF-binding sites can disrupt chromatin looping and alter the expression of target genes [23, 27]. Methods to predict chromatin-interaction frequencies and/or topological-associated domains from large, genome-wide epigenetic datasets [28, 29] have been proposed, but the broader role of DNA methylation in mediating 3D organization of the genome remains poorly understood.

Here, we developed a new method, which is based on the information from multiple samples, to identify functional UMCs. We define sparse conserved under-methylated UMC (scUMC) as CpG maintained at under-methylated levels and sparsely distributed in highly methylated background in multiple cell types. The scUMCs are found in distal anchor regions co-occupied by multiple chromatin-loop factors (RAD21, SMC3, CTCF, and ZNF143). Despite the fact that neighboring CpGs are highly methylated, the binding intensity of chromatin-loop factors and interaction frequencies associated with scUMC are comparable to those observed with conventional, long UMRs. Furthermore, cell-type-specific methylation of scUMCs (such as during cell lineage commitment) is concomitant with reduced chromatin interactions and chromatin-loop factor binding and altered gene expression programs. Overall, our results demonstrate that a new epigenetic feature, scUMC, is involved in cell-specific regulation of long-range chromatin interaction mediated by chromatin-looping factors.

## Results and discussion

### Identification of sparse under-methylated CpG conserved across cell types

**Fig. 1:**
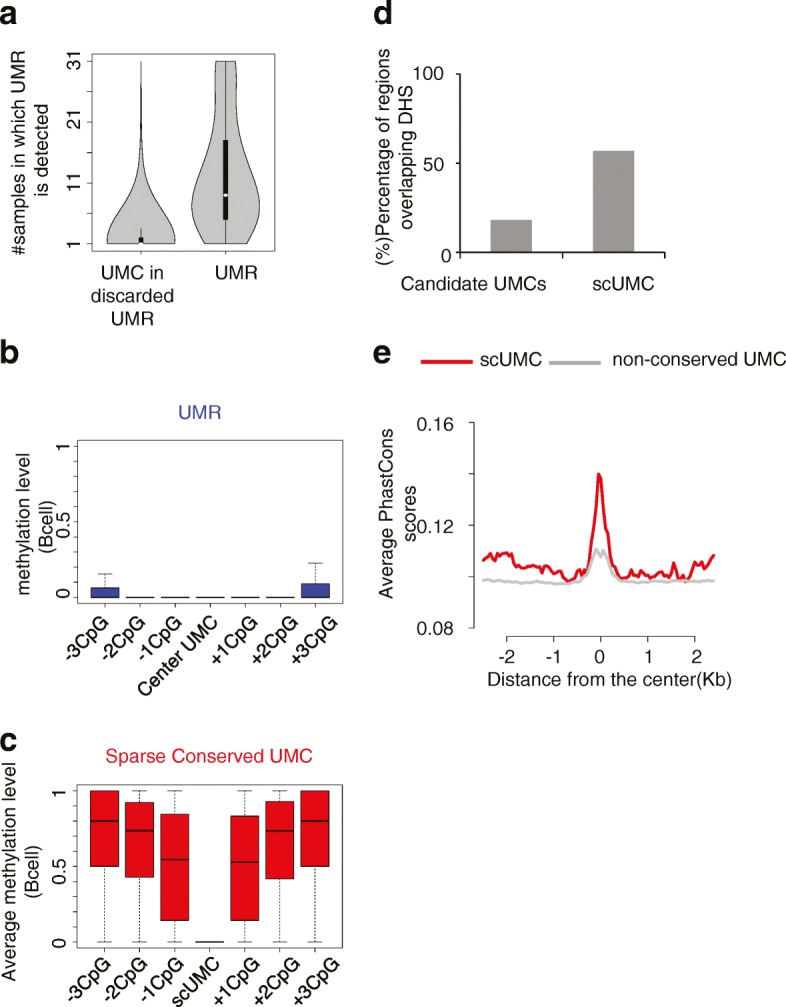
Identification of scUMC. **a** Number of sample methylomes sharing either a given UMR (no less than four CpG, *right*) or a given UMC in an otherwise discarded UMR (less than four CpG, *left*). **b** Methylation levels of central UMC and flanking CpG sites in UMRs detected in B cells. **c** Methylation levels of scUMCs and flanking CpG sites detected in B cells. **d** Percentage of indicated epigenetic features (candidate UMCs or scUMCs) overlapping DHSs. Candidate UMCs represent all UMCs in discarded UMRs from the population of methylomes (n = 31). Further details are provided in “Methods” and Fig S2. **e** Average vertebrate PhastCons scores in 2.5-kb region flanking scUMCs or non-conserved UMCs (Chebyshev’s Inequality Probability < 0.95; see “Methods” for further details)

Compared with long UMRs (regions including ≥ 4 UMCs), the majority of single-base UMCs in a methylome population are sample-specific (~93%) (Fig. 1a). Thus, the first step in detecting functional UMC is to remove those which occur stochastically. The recent adoption of WGBS by the epigenetics field has led to a number of high-quality reference methylomes. We utilized the information from a large number of biological replicates to quantify single-base UMC frequency in the population. Our method is as follows (Additional file 1: Figure S2): first, we collected all the UMCs from 31 phenotypically normal human cell model WGBS datasets passing stringent quality controls and processed via the same analysis workflow. We removed sites in long UMRs as well as those associated with SNPs. Second, we assigned an under-methylation conservation score to each candidate UMC based on the observed frequency in the population. Third, we selected a set of conserved UMCs based on modeling the scores according to Chebyshev’s Inequality [30], a robust, probabilistic method to detect outliers without assumption of the distribution. When applying the cutoff *p* < 0.01, only those UMCs conserved in ten or more methylomes are designated functional UMCs (Additional file 1: Figure S2). Our analysis identified 9421 UMCs satisfying these criteria. Whereas spatially proximal UMCs cluster together to form conventional UMRs (Fig. 1b), our predicted functional UMCs are flanked by highly methylated CpGs (Fig. 1c). Further, they are enriched in open, DNase I-accessible chromatin (Fig. 1d) and evolutionarily conserved (Fig. 1e). Given their characteristics, (1) sparse distribution in a highly methylated background, (2) DNase I hypersensitivity, (3) evolutionary and (4) epigenetic conservation (maintaining under-methylated states in multiple cell types), we termed them scUMC. Detailed comparisons between scUMC and almost all (to the best of our knowledge) previous studies for UMRs demonstrate that scUMC is indeed a novel epigenetic feature with negligible overlap with previously reported UMRs. (Additional file 1: Figure S1d; Additional file 2: Table S3 and S4).

### scUMCs are enriched in chromatin-loop factors and long-range chromatin interactions

**Fig. 2:**
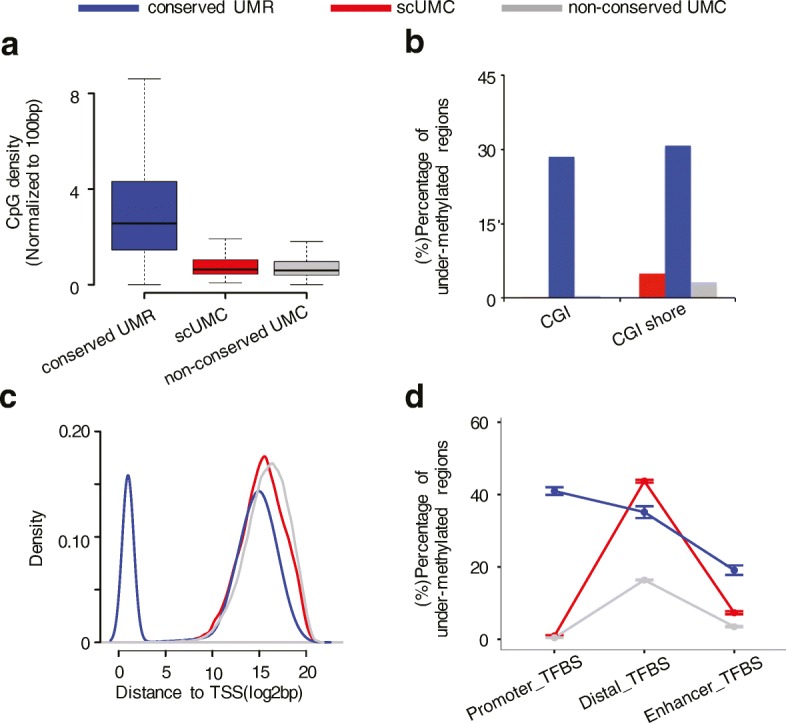
Features of scUMCs. **a**–**d** Three groups of under-methylated features are color-coded: *blue* (conserved UMRs), *red* (scUMCs), and *gray* (non-conserved UMCs). **a** CpG density (normalized to 100 bp). **b** Percentage of features associated with either CGI or CGI shore. **c** Distribution of distances to TSSs. **d** Percentage of features associated with regulatory elements from ENCODE. Promoter and enhancer regions are defined by chromatin state segmentation (ChromHMM) from ENCODE as described in “Methods.” TFBSs are ChIP-seq peak clusters for 161 transcription factors (ENCODE). Promoter TFBS is the subset of TFBSs with overlapping promoter states. Enhancer TFBS is the subset of TFBSs with overlapping enhancer states. Distal TFBS is the subset of TFBS not overlapping chromHMM Promoter or Enhancer states. The error bar is the 95% confidence interval of percentage for nine cell lines involving chromatin state segmentation

If 9421 scUMCs are functionally distinct from 43,996 conserved UMRs (see “Methods”), we reasoned they should differ in CpG density and proximity to gene promoters. In contrast to conserved UMRs, scUMCs are markedly CpG-poor (Fig. 2a) and generally not found in either CpG islands [31] or CpG island shores (Fig. 2b). The scUMCs occur distal to transcriptional start sites (TSSs) whereas conserved UMRs are equally likely to be found proximal or distal (Fig. 2c). To investigate scUMCs further, we considered functional elements predicted by an unbiased, data-driven approach: chromatin state segmentation [9]. As expected, the analysis found scUMCs are not associated with promoter states (Additional file 1: Figure S3), but are enriched in insulator elements compared to conserved UMRs. The scUMCs are associated with enhancer states, but to a much lesser extent than conserved UMRs. Next, we examined the relationships between scUMCs and peak clusters of DNA binding for 161 transcription factors in 91 cell types from the ENCODE Project Consortium [9]. The scUMCs show strong enrichment for distal TFBSs not overlapping with either promoters or enhancers, whereas conserved UMRs are similarly enriched for both promoter and distal TFBSs (Fig. 2d).

**Fig. 3:**
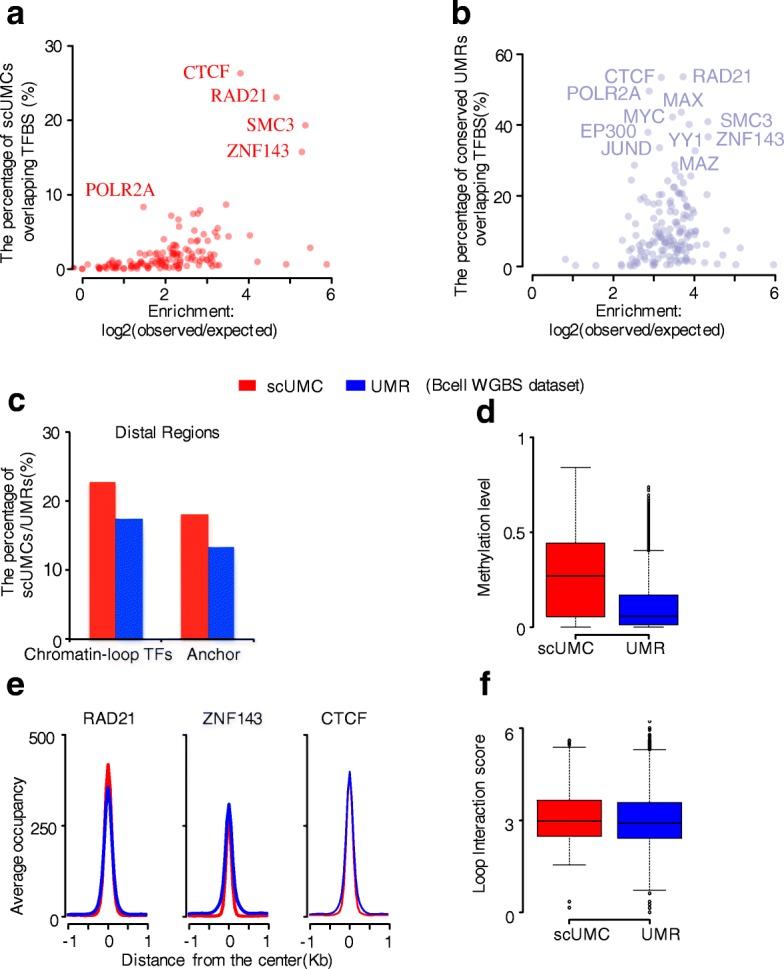
scUMCs are enriched in chromatin interactions. **a**, **b** scUMC (**a**) and conserved UMR (**b**) enrichment for 161 transcription factors (TF); the *x-axes* represent the log2 ratio of observed vs. expected number of features overlapping each TF; the *y-axes* represent the percentage of features bound by each TF. For a more accurate comparison with the genome-wide distribution of scUMCs, distal conserved UMRs (greater than ± 3 kb from TSS) are plotted (**b**). **c** Percentage of distal epigenetic features (*red*, scUMC; *blue*, UMR) in B cells overlapping chromatin-looping factors or anchors of chromatin interactions. Chromatin-looping factor sites represent distal regions (greater than ± 3 kb from TSS) co-occupied by Rad21, Znf143, and CTCF ChIP-seq peaks in GM12878 B cells (ENCODE). Anchors represent chromatin-looping interactions of Rad21 measured by ChIA-PET in GM12878 B cells [14]. **d** Average methylation level in B cells of chromatin-looping factor sites containing either scUMCs or UMRs. **e** Average occupancy of chromatin looping factors (Rad21, Znf143, and CTCF) centered on either scUMCs or UMRs in B cells. **f** Interaction intensity of anchor regions overlapping either scUMCs or UMRs. The loop interaction score is as published in Heidari et al. [14]

Having established that scUMCs are associated with distal TFBSs collectively, we next asked whether the relationship was characterized by enrichment for particular DNA-binding proteins. We identified four factors specifically enriched in scUMCs, including RAD21, SMC3, CTCF, and ZNF143 (Fig. 3a; Additional file 1: Figure S4a). Enrichment of each factor is present but considerably reduced in conserved UMRs (*p* value = 0.045; one-tailed t-test) by comparison and indistinguishable from other TFs such as POLR2A, MAX, MYC, YY1, and EP300 (Fig. 3b). These four factors (RAD21, SMC3, CTCF, and ZNF143) are present at the anchor regions of chromatin interactions, serving as chromatin-looping factors [14]. To investigate this relationship further, we focused on looping factor occupancy and chromatin-interaction frequency at scUMCs in a particular cell type. The GM12878 is a well-characterized cell model for the lymphoid-committed B-cell lineage. We obtained published looping factor ChIP-seq and chromatin interactions detected by RAD21 ChIA-PET in GM12878 cells. We compared these datasets to predicted scUMCs and conserved UMRs (5237 and 43734) in B cells (GSM791827 [32]). The scUMCs show increased enrichment both for sites co-occupied by looping factors as well as distal interacting anchors compared to conserved UMRs (Fig. 3c). The scUMCs in chromatin-loop factor binding sites are proximal to highly methylated CpG, resulting in marked differences between the average methylation levels of binding sites with scUMCs compared to sites with conserved UMRs (Fig. 3d). Nevertheless, the binding intensities of chromatin-loop factors (Rad21, Znf143, and CTCF) to regions with scUMCs or with UMRs are quite comparable (Fig. 3e). Rad21 interaction frequencies are slightly greater for anchor regions associated with scUMCs compared to UMRs (Fig. 3f). These central findings regarding scUMCs, comparable (1) intensity and (2) methylation level of chromatin-loop factor binding sites, and (3) cohesin subunit interaction frequencies were replicated in independent analyses of H1 embryonic stem cells (ESCs) (Additional file 1: Figure S4b–f). Thus, several lines of evidence suggest scUMCs are characteristic of distal functional genomic elements and distinct from conserved UMRs. scUMCs are more frequently associated with looping factor occupancy as well as anchor regions of chromatin loops. Even though the regions are more highly methylated as a whole, they show the same level of factor occupancy or interaction frequency compared to regions with conserved UMRs.

### Methylation of scUMCs impacts chromatin-loop factors occupancy and the intensity of chromatin interactions

**Fig. 4:**
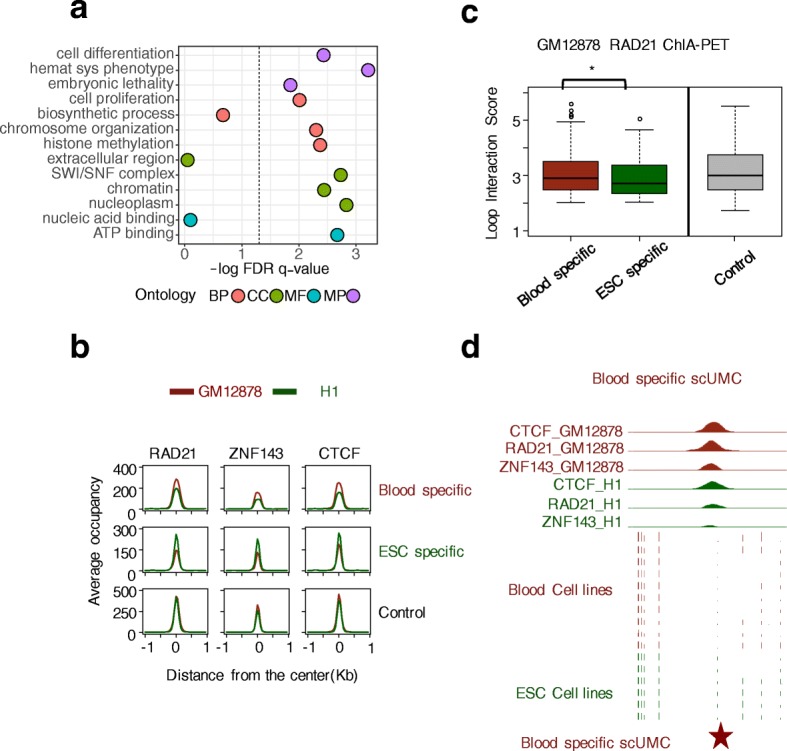
Cell lineage scUMCs are associated with the dynamic regulation of chromatin loops. **a** Processes and functions enriched for cell lineage-specific scUMCs. Functional significance of scUMCs differentially methylated between ESCs and blood cell lineages were predicted by GREAT 2.0. *Y-axis* represents the log-transformed, FDR-corrected hypergeometric *p* value. Ontology sources are color-coded: Gene Ontology Biological Process (BP), Cellular Component (CC), Molecular Function (MF), and Mouse Genome Informatics Phenotype (MP). Associations in GREAT based on gene-regulatory domain basal (±3 kb TSS) plus up to 500-kb extension. **b** Dynamic occupancy of chromatin-looping factors (Rad21, Znf143, and CTCF) in GM12878 (*coral*) and H1 (*green*) cells at regions centered on scUMCs: blood-specific, ESC-specific, or control. **c** Distribution of Rad21 chromatin-looping interaction intensities in GM12878 cells [14] for anchor regions overlapping scUMCs: blood-specific, ESC-specific, or control. *P value < 0.05, Wilcoxon signed-rank test, one-tail. **d** Representative genomic region of a blood-specific scUMCs. *Top*, ChIP-seq signal densities of Rad21, Znf143, CTCF in GM12878 and H1 cells. *Bottom*, CpG methylation ratios in ESCs or blood-lineage cells

If scUMCs play a functional role in mediating higher order chromatin interactions, we reasoned that gain of methylation would perturb chromatin interactions. ESC and blood-cell lineages are similarly represented in the population of methylomes we used to define scUMCs and can be clearly clustered into two groups by methylation level (Additional file 1: Figure S5). Further, they model a critical cell-fate decision point in stem-cell biology. Methylation differences could reflect biological differences. Thus, we compared the methylation levels of scUMCs in chromatin-looping factor binding sites (n = 2195) between the two groups, ESCs and cells committed to the blood lineage. We identified 177 and 285 scUMCs specific to ESCs and blood cells, respectively (Additional file 1: Figure S6). Next, we asked whether the differentially methylated scUMCs were associated with particular types of genes or biological functions. We found cell lineage-defining scUMCs are associated with essential developmental genes, regulators of cell differentiation as well as hematopoietic system phenotypes (supported by mouse knockout models). Interestingly, they also include nuclear proteins with specific functions in chromosome organization, including chromatin remodeling (SWI/SNF) and histone methylation (Fig. 4a). The epigenetic changes clearly reflect and are consistent with stem-cell differentiation and commitment to the functional blood-cell lineage. We then investigated whether cell-specific scUMCs reflected differences in binding of chromatin-loop factors (Rad21, Znf143, and CTCF) between GM12878 blood and H1 ESCs. Factor binding at blood-specific scUMCs was significantly decreased in H1 ESCs and conversely, binding at ESC-specific scUMCs was significantly reduced in GM12878 cells (Fig. 4b and Additional file 1: Figure S7a). Binding was not affected at scUMCs common to both cell types. These results indicate the methylation state of scUMCs can be directly related to the binding intensity of chromatin-loop factors. To test the impact of cell-specific scUMCs on functional chromatin interactions, we compared ChIA-PET experiments of Cohesin complex members RAD21 [14] in GM12878 blood and SMC1 [17] in H1 ESCs (to the best of our knowledge, GM12878 Rad21 and H1 SMC1 are the only two suitable ChIA-PET datasets that also have corresponding WGBS methylation data). In GM12878 cells, RAD21 interaction intensity is increased for loop anchor regions with blood-specific scUMCs compared to regions with ESC-specific scUMCs (Fig. 4c, d). Conversely, SMC1 interaction intensity in H1 cells is increased for loop anchor regions with ESC-specific compared to blood-specific scUMCs (Additional file 1: Figure S7b). Furthermore, in the additional analysis between blood lineage and alternate cell commitment fibroblast/neuron lineages, we again observed the increase of RAD21 interaction in loop anchor regions with blood-specific scUMCs compared to regions with fibroblast/neuron-specific scUMCs, although with a *p* value (0.057) trending towards significance (Additional file 1: Figure S8). In summary, despite the fact that scUMCs represent individual unmethylated CpG among a highly methylated background, scUMCs’ gain of methylation is directly linked to both weakened binding of chromatin-loop factors as well as reduced chromatin interactions (mediated by the chromatin-loop factors).

### scUMC dynamics and gene expression

**Fig. 5:**
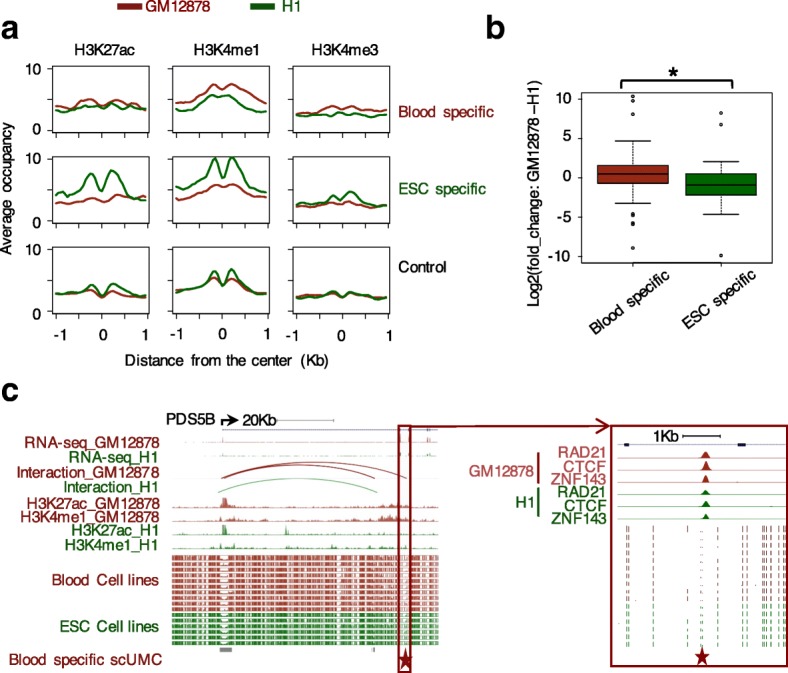
scUMC dynamics and gene expression. **a** Dynamic occupancy of activating histone marks in in GM12878 (*coral*) and H1 (*green*) cells at regions centered on scUMC: blood-specific, ESC-specific, or control. **b** Distribution of expression differences (GM12878 – H1) among target genes of blood-specific and ESC-specific scUMCs. *p value < 0.05, Wilcoxon signed-rank test, one-tail. **c** Representative genomic region of blood-specific scUMC target gene *PDS5B*. Tracks include RNA sequencing (RNA-seq) and H3K27ac, H3K4me1 ChIP-seq signal densities for GM12878 and H1 cells; chromatin interactions, Rad21 ChIA-PET for GM12878 and SMC1 for H1; CpG methylation ratios in ESCs or blood-lineage cells. The scUMC is highlighted in a greater magnification window along with Rad21, CTCF, and Znf143 ChIP-seq signal densities for GM12878 and H1 cells

Chromatin loops anchored by Rad21, Znf143, and CTCF are known to bridge the enhancer and promoter and regulate gene expression [14, 15]. Although scUMCs do not overlap enhancer regions with the same frequency as conserved UMRs (Additional file 1: Figure S3), they are more enriched for the anchors of chromatin-interaction loops. Histone modifications, such as H3K27ac and H3K4me1, can also contribute to the cell-specific binding and interactions [14, 33]. We asked whether cell-specific scUMCs are associated with distinct patterns of histone modifications. Interestingly, we observed cell-specific increases in the profiles of active enhancer marks, such as H3K27ac, H3K4me1 centered on cell-specific scUMCs, but not for inactive mark H3K27me3 (Fig. 5a, Additional file 1: Figure S9a). The active marks are depleted at the sites of scUMCs but enriched around their flanking regions, suggesting the scUMCs are found in nucleosome-free DNA.

Next, we investigated whether cell-specific scUMCs in chromatin loops are associated with cell-specific gene expression programs. We used the anchors of chromatin loops to associate scUMCs with scUMC-target genes. If the mate of an anchor region containing a scUMC overlapped with the promoter region (±3 kb from TSS) of a gene, it was considered the scUMC-target gene. We used ChIA-PET experiments of Cohesin complex members RAD21 [14] in GM12878 blood and SMC1 [17] in H1 ESCs to detect blood- and ESC-specific target genes separately (Additional file 1: Figure S9b). The control scUMC-target genes were the combined results of the two (Additional file 1: Figure S9b). Analysis of GM12878 and H1 RNA-seq profiles indicate cell-specific scUMC-target genes, as a population, tend to be more highly expressed in the cell type of which they are defined (Fig. 5b). One blood-specific scUMC-target gene of interest is *PDS5B*. The yeast homolog *Pds5* functions as a regulatory subunit of the Cohesin complex. Human *PDS5B* has been shown to be a negative regulator of cell proliferation and may function as a tumor suppressor [34]. In GM12878 cells, a RAD21 interacting loop bridges the *PDS5B* promoter and blood-specific scUMCs together with a specific enhancer beside this scUMC, resulting in higher expression of *PDS5B* in GM12878 cells (GM12878 vs. H1 rpkm values: 9.37 vs. 3.31; *p* value < 0.05) (Fig. 5c). Collectively these results suggest that cell-type-specific scUMCs correlate with differential gene expression by impacting chromatin high-order structure and interactions.

## Conclusion

In general, sparse UMCs are discarded by conventional approaches for predicting functional UMRs. Our multi-sample-based method identifies a novel epigenetic feature, scUMC, whose functionality is suggested by multiple lines of experimental evidence: DNA binding of chromatin-looping factor, chromatin-interaction intensity, and gene expression. We demonstrate evidence of functionality in both ESCs as well as blood lineage-committed cells and that differential methylation of scUMCs reflects their biological differences, being significantly enriched for genes involved in stem-cell differentiation and hematopoietic phenotypes. Despite the fact scUMCs represent individual unmethylated cytosines among a highly methylated background, scUMC gain of methylation is directly linked to both weakened binding of chromatin-loop factors as well as reduced chromatin interactions. In fact, much of the variation in CTCF binding has been linked to differential DNA methylation, concentrated at two critical positions within the CTCF recognition sequence [35]. We are only now beginning to understand the role of gene distal methylation alterations in disease [36, 37]. Disruption of a topological domain boundary by DNA methylation upregulates the oncogene *PDGFRA* in IDH mutant gliomas [23]. We observed roughly 15% of scUMCs occur in such boundaries delineated by ChIA-PET [24]. Therefore, further studies of the role scUMCs may play in boundary collapse or other aberrant chromatin interactions during tumorigenesis are warranted.

## Methods

### Published datasets

In this study, we used a total of 51 datasets (Additional file 3: Table S5) including 31 WGBS, 16 ChIP-seq, two ChIA-PET, and two RNA-seq obtained from Roadmap Epigenomics and ENCODE. CpG island (CGI) reference coordinates were downloaded from the UCSC genome annotation database. DHS clusters, peak clusters of 161 TFBS (wgEncodeRegTfbsClusteredV3), wgEncodeBroadHmm tables were generated by the ENCODE Project Consortium and downloaded from the UCSC database. The wgEncodeBroadHmm datasets represent chromatin state segmentation for nine human cell types learned by computationally integrating ChIP-seq data for nine factors plus input using a HMM [38]. Promoter and Enhancer states presented in Fig. 2d represent the union of multiple states describing these same broader categories. Analyses including all predicted states for all nine cell types are presented in Additional file 1: Figure S3.

### WGBS data pre-processing

For each WGBS sample in 31 normal cell types (Additional file 3: Table S5), we use BSMAP to trim adaptor and low-quality sequences with default threshold, align bisulfite-treated reads to human hg19 genome. BSeQC [39] was then used to remove the technical biases in WGBS data, introduced by end repair, polymerase chain reaction (PCR) amplification, and overlapping segments in paired-end reads. We used MOABS [40] to calculate the methylation ratio for CpG sites supported by at least four aligned reads.

### UMR detection

The UMRs are identified with the requirement of at least four consecutive hypomethylated CpG sites and a mean methylation ratio < 10% as previously described [5]. A total of 1,397,217 UMRs were identified from the 31 samples.

### Conservation score

First, we collected all UMCs (UMC: %mCpG ≤ 10%) found in at least one sample methylome. Next, we excluded UMCs lying in conventional UMRs from the subsequent analysis. In addition, the UMCs lying in SNPs are also removed. The result is candidate UMCs that may be scUMCs. Finally, we used the following formula to calculate the conservation score for a candidate UMC.

$$ {\displaystyle \begin{array}{c}\hfill S=\frac{\sum_{i=1}^N{s}_i}{N}\times 100\hfill \\ {}\hfill where\ {s}_i=\left\{\begin{array}{c}\hfill 1, if\ s\  is\ a\  UMC\  in the\  ith\  sample\hfill \\ {}\hfill 0,\kern0.5em otherwise\ \hfill \end{array}\right.\hfill \end{array}} $$

### Sparse conserved under-methylated CpG (scUMC) detection

It is expected the UMC with a higher conservation score (occurs in more samples) is more likely to be a functional region. Therefore, credible UMC detection is essentially the identification of “outlier” UMCs with significant conservation scores. Here, we used Chebyshev’s Inequality to detect the credible UMCs and merging regions within 300 bp to obtain scUMCs. Chebyshev’s Inequality is a non-parametric method to detect outliers [30]. This method is statistically robust and does not make assumptions of the distribution of UMC conservation scores. Chebyshev’s Inequality is usually stated for a random variable. Let *X* has a finite mean *μ* and finite non-zero variance *σ* ^*2*^, then for any real number *k* > 1:

$$ \boldsymbol{\Pr}\left(|\boldsymbol{X}-\boldsymbol{\mu} |\ge \boldsymbol{k}\boldsymbol{\sigma } \right)\le \frac{1}{{\boldsymbol{k}}^2} $$

Applying this into credible scUMC detection and using as *m* and *v* as estimators of *μ* and *σ* ^*2*^. For UMC conservation score, *m* and *v* are its mean and standard deviation in all the WGBS data. So, we have:

$$ \boldsymbol{\Pr}\left(|\boldsymbol{X}-\boldsymbol{m}|\ge \boldsymbol{kv}\right)\le \frac{1}{{\boldsymbol{k}}^2} $$

For a UMC, if its conservation score in all the WGBS data was 4.5 times of *v* (standard deviation) larger than the *m* (mean), then the probability of finding a UMC which occurs with the same or more samples than this UMC in these 31 WGBS data is 1/(4.5**2) ≈ 0.05. This value is a kind of *p* value. Based on this criterion of *p* = 0.01, only the candidate UMCs conserved in ten or more samples would be detected as scUMCs. Non-conserved UMC is a subset of candidate UMCs based on non-significant *p* value (0.95) without scUMC.

### Conserved under-methylated region (UMR) detection

In order to compare scUMCs, we identified the conserved UMRs from all the UMRs detected from the 31 high-quality methylomes. We merged a total of 1,397,217 UMRs to 260,150 non-redundant UMRs. We then calculated the conservation score as described above for each UMC lying inside the non-redundant UMR. We defined 43,996 conserved UMRs with at least one UMC lying inside the UMR with the same conservation cutoff (not less than ten samples).

### FDR calculation for minimal number of CpG in UMR

To calculate the FDR for a cutoff of the minimal CpG number in a conventional UMR, we compared the UMR detected in the original methylome with a randomized methylome by HMM. For a given methylome, we performed a random shuffle for the methylation level of all the CpGs to destroy the spatial correlation in nearby CpGs and construct the randomized methylome. Thus, we detected the UMR in the randomized methylome by the same procedure. The resulting null distribution indicates the minimal CpG number required in classic UMR detection.

### Rad21, CTCF, Znf143, and histone modification ChIP-seq data analysis

The raw reads for ChIP-seq data were downloaded from Gene Expression Omnibus and the detail information about the data were listed in Additional file 3: Table S5 [41]. Reads were mapped to human genome hg19 using BWA [42]. Reads that could be mapped to multiple locations were removed. To remove the PCR resulted clonal reads, two clonal reads at the most were kept for subsequent analysis. The number 2 was based on Poisson *p* value cutoff of 1 × 10 − 5 determined by the total number of reads with respect to the theoretical mean coverage across the genome. Then, the remaining reads were analyzed with DANPOS v2.2.1 [43] for read depth normalization, input signal subtraction, and occupancy calculation.

### RNA-seq data analysis

Raw reads for GM12878 (GSM958728;GSM958742) and H1 (GSM958733;GSM958743) cells were downloaded from Gene Expression Omnibus [44]. We used Trim Galore (http://www.bioinformatics.babraham.ac.uk/projects/trim_galore/) to trim the low-quality bases and the adapters. TopHat [45] was used to mapping the raw reads on hg19 with the default parameters. The gene annotation used for transcriptome alignment is hg19 GTF annotation file from UCSC annotation database. Differentially expressed genes were defined by the cutoff: FDR ≤ 0.05 using the function Cufdiff in Cufflinks [46].

## Additional files

Additional file 1: A PDF file containing all supplementary figures. (DOCX 1444 kb)

Additional file 2: A.docx file containing Tables S1–S4. (DOCX 282 kb)

Additional file 3: Table S5. is an.xls file containing all datasets (31 WGBS, 16 ChIP-seq, two RNA-seq, and two ChIA-PET) used in this study. (XLS 36 kb)

Additional file 4: Table S6. is an.xls file containing 9421 scUMCs detected in 31 WGBS. (XLS 1919 kb)

## Abbreviations

ChIP-seq: Chromatin immunoprecipitation followed by high-throughput DNA sequencing
ChromHMM: Software for learning and characterizing chromatin states
DHS: DNase hypersensitivity site
DMV: DNA methylation valley
ENCODE: Encyclopedia of DNA Elements
ESC: Embryonic stem cell
HMM: Hidden Markov Model
HMR: Hypo-methylated region
LMR: Low methylated region
RNA-seq: RNA sequencing
scUMC: Sparse conserved under-methylated CpG
TF: Transcription factor
TFBS: Transcription factor binding site
UMC: Under-methylated CpG
UMR: Under-methylated region
WGBS: Whole-genome bisulfite sequencing
