# Supplementary material for: Sparse conserved under-methylated CpGs are associated with high-order chromatin structure
Source: Genome Biol. 2017 Aug 31;18:163. doi: 10.1186/s13059-017-1296-x (PMC5580327; doi:10.1186/s13059-017-1296-x)
Supplement: Supplementary file 1 — A PDF file containing all supplementary figures. (DOCX 1444 kb) [file 13059_2017_1296_MOESM1_ESM.docx]

**Supplementary Figures for Sparse Conserved Under-methylated CpG is associated with** **high-order chromatin structure**

**Supplemental Figures**

**Figure S1: UMR and LMR predictions in H1 ESC WGBS dataset.**

**(a)** Barplot of the False Discovery Rate (FDR: calculation described in Methods section) associated with UMR predictions requiring an increasing minimal number of CpG. UMR prediction was performed on the methylome of H1 cells with MethylSeekR R package implemented in R. **(b)** Percentage of UMR in H1 cells overlapping DNAse Hypersensitive Sites (DHS) as a function of the number of CpG sites in UMR. **(c)** Barplot indicating the absolute number of UMR in H1 cells overlapping DHS as a function of the number of CpG sites in UMR.

**(d)** Venn diagram of overlap between scUMC and low-methylated regions (LMR) predicted in H1 cells. LMR were predicted by MethylSeekR R package.

**Figure S2: Graphical overview of workflow detecting sparse conserved under-methylated CpG**

**(a)** Step 1, 31 WGBS datasets were uniformly processed. Step 2, detection of conserved UMRs (regions including >= 4UMCs) and all the UMCs (%mCpG<=10%) in 31 samples. Step 3, remove UMCs located in classic UMRs and SNP and gain the candidate UMCs. Step 4, calculate the conservation score based on the observed frequency in 31 samples. Step 5, identify 9421 sparse conserved UMC based on Chebyshev’s Inequality.

**Figure S3: Distribution of epigenetic features in regulatory elements summarized across multiple cell types.**

**(a)** The distribution of regions of interest (conserved UMR, scUMC and UMC_NotCon) in different regulatory chromatin states. The states are taken from ENCODE chromatin state segmentation for nine human cell types learned by computationally integrating ChIP-seq data for nine factors plus input using a Hidden Markov Model[^1^](#_ENREF_1). The files representing 15 chromatin states defined in 9 cell types were obtained from the UCSC genome annotation database. Data are faceted by chromatin state and the distributions of overlap across cell types with our regions of interest (percentage of regions) are plotted.

**Figure S4: TFBS enrichment for non-conserved UMC and scUMC in H1 ESC**

**(a)** Enrichment of non-conserved UMC (see Method for further detail) in binding site clusters for 161 transcription factors (TF) defined by ENCODE; the x-axis represents the log2 ratio of observed versus expected number of features overlapping each TF; the y-axis represents the percentage of features bound by each TF. **(b)** Average methylation level in H1 cells of chromatin-loop factor sites containing either scUMC or UMR. **(c)** Interaction intensity of cohesin complex subunit SMC3 ChIA-PET anchor regions overlapping either scUMC or UMR in H1 cells. The loop Interaction score is as published in Ji X et al. 2016. **(d)** Average occupancy of chromatin looping factors (Rad21, Znf143 and CTCF) centered on either scUMC or UMR in H1 cells. **(e)** Representative genomic region of an ESC-specific scUMC. Top, ChIP-seq signal densities of Rad21, Znf143, CTCF in GM12878 and H1 cells. Bottom, CpG methylation ratios in ESC or blood-lineage cells. (f) The categories of scUMC associated CTCF (green: H1; coral: GM12878). The TAD domains are downloaded from the 3D Genome Browser website: http://promoter.bx.psu.edu/hi-c/publications.html.

**Figure S5:** Clustering of all the scUMCs (9142) on their DNA methylation profiles shows tissue specificity. 31 cell lines (columns) were cluster into five major groups. The cell lines inside each group are as follow:

**ESC:** H1 Derived Mesenchymal Stem_Cells; H1 BMP4 cell line; H1 BMP4 Derived Mesendoderm CulturedCells; ESC_1; ESC_2; H1; H9; Wa09; hESC_Derived_CD56+_Ectoderm_Cultured_Cells_3; hESC_Derived_CD56+_Ectoderm_Cultured_Cells; hESC_Derived_CD56+_Ectoderm_Cultured_Cells_2; HUES64_2; HUES64.

**Blood:** CD56 Primary Cells; B Lymphocyte (Bcell); CD19 Primary Cells; CD3 Primary Cells; pbmc; HSC CD133; CD14 Primary Cells; CD34 Primary Cells; Hematopoietic stem cells; neutrophil.

**Neuron:** ganglionic eminence derived_free1; neurosphere cultured cells, ganglionic eminence derived_free2; neurosphere cultured cells, cortex derived_2; neurosphere cultured cells, cortex derived_1.

**Fibroblast:** IMR90_1; IMR90_2; Wa09fibro.

**HMEC**

**Figure S6:** Workflow to define the three groups of scUMC: ESC-specific, Blood-specific, and Control. (The detail information for WGBS data is in the TableS1).

**Figure S7: ESC-specific scUMC have stronger binding of chromatin-loop factors and increased loop interaction in H1 ESC**

**(a)** Representative genomic region of an ESC-specific scUMC. Top, ChIP-seq signal densities of Rad21, Znf143, CTCF in GM12878 and H1 cells. Bottom, CpG methylation ratios in ESC or blood-lineage cells. **(b)** Distribution of SMC1 chromatin looping interaction intensities in H1 cells (Ji X et al. 2016) for anchor regions overlapping scUMC: blood-specific, ESC-specific, or control. The loop interaction score is as published in Ji X et al. 2016. *, p-value <0.05, Wilcoxon signed-rank test, one-tail.

**Figure S8: Cell lineage scUMC are associated with the dynamic regulation of chromatin loops between blood lineage and alternate cell commitment Fibroblast/Neuron lineages (a)** Table for cell specific scUMCs relative to blood lineage. **(b)** Distribution of Rad21 chromatin looping interaction intensities in GM12878 cells (Heidari N et al. 2014) for anchor regions overlapping scUMC: blood-specific, Fibroblast/Neuron specific. p-value= 0.057, Wilcoxon signed-rank test, one-tail.

**Neuron:** neurosphere cultured cells, ganglionic eminence derived_free1; neurosphere cultured cells, ganglionic eminence derived_free2; neurosphere cultured cells, cortex derived_2; neurosphere cultured cells, cortex derived_1.

**Fibroblast:** IMR90_1; IMR90_2; Wa09fibro.

**Figure S9: Data supporting Figure 5**

**(a)** Dynamic occupancy of H3K27me3 in in GM12878 (coral) and H1 (green) cells at regions centered on scUMC: blood-specific, ESC-specific, or control. **(b)** Barplot indicating the number of predicted target genes for scUMC: blood-specific, ESC-specific, or control.

1 Ernst, J. *et al.* Mapping and analysis of chromatin state dynamics in nine human cell types. *Nature* **473**, 43-49 (2011).

2 "The 3D Genome Browser: a web-based browser for visualizing 3D genome organization and long-range chromatin interactions." <http://biorxiv.org/content/early/2017/02/27/112268>, Biorxiv, 2017.
