# Supplementary material for: Sparse conserved under-methylated CpGs are associated with high-order chromatin structure
Source: Genome Biol. 2017 Aug 31;18:163. doi: 10.1186/s13059-017-1296-x (PMC5580327; doi:10.1186/s13059-017-1296-x)
Supplement: Supplementary file 2 — A.docx file containing Tables S1–S4. (DOCX 282 kb) [file 13059_2017_1296_MOESM2_ESM.docx]

**Table S1. Literature review for the co-occupancy of four factors (RAD21, SMC3, CTCF, and ZNF143) on anchors of chromatin-loop.**

| **Reference** | **Conclusions and Figures(Tables)** |
| --- | --- |
| Heidari et al., 2014 | RAD21, SMC3, CTCF, and ZNF143 were strongly enriched at interacting location(anchor; defined by ChIA-PET);  Figure 2A |
| Branch et al., 2013 | Computational analysis on ChIP-seq datasets from ENDODE revealed that (1) most constitutive CTCF often co-localized with constitutive cohesin loci and constitutive Znf143 loci; (2) most constitutive CTCF loci were present in CTCF-mediated chromatin interactions detected by ChIA-PET.  Table S1; Table 3 |

**Table S2. Literature review for the causal relationship between RAD21/SMC3/CTCF/ZNF143 and chromatin interaction**

| **Reference** | **Conclusions and Figures** |
| --- | --- |
| Splinter et al. 2006 | Deletion of CTCF destabilized long-rang interactions and local histone modification.  Figure 1 |
| Hadjur et al. 2009 | RAD21 knockdown substantially reduced long-range interactions of INFG coding region.  Figure 3 |
| Hou et al. 2010  (Cell type specificity of chromatin organization mediated by CTCF and cohesion) | Reduction of CTCF or Rad21 results in loss of long-range interactions.  Figure 2 and Figure3 |
| Sofueva et al. 2013  (Cohesin-mediated interactions organize chromosomal domain architecture.) | Loss of a functional cohesion complex (Rad21) perturbs nuclear structure.  Figure 2 |
| Phillips-Cremins et al. 2013 | Knockdown of Smc1 or Med12 in embryonic stem cells results in disruption of spatial architecture and downregulation of genes found in cohesin-mediated interactions. (In cells, SMC1 and SMC3 are believed to always exist as heterodimers in a one-to-one stoichiometry).  Figure 5 and Figure 6 |
| Zuin et al. 2014  (Cohesin and CTCF differentially affect chromatin architecture and gene expression in human cells) | Deletion of CTCF or Rad21 reduces the chromatin interactions.  Figure 2 and Figure 3 |
| Sanborn et al. 2015 | Genome editing of CTCF motifs disrupts corresponding loops.  Figure 7 |
| Guo et al. 2015 | CRISPR inversion of CTCF sites alters long-range chromatin-looping interactions.  Figure 4 |
| Bailey et al., 2015 | Silencing ZNF143 or disruption of ZNF143 binding by genetic variance decreased the frequency of chromatin interaction. |

**Table S3 Comparison with other Hypo-methylated regions**

| **DNA methylation Regions** | **Length** | **Average methylation level** | **Method** | **% scUMCs (H1 cell line)** | **Reference** |
| --- | --- | --- | --- | --- | --- |
| **DNA methylation valleys (DMV)** | >= 5Kb | < 15% | Window-based Method | 0.15% | Xie et al., 2013 |
| **DNA methylation Canyon** | >= 3.5Kb | <10% | Two states HMM | 0.21% | Jeong et al., 2014 |
| **Hypo methylation region (HMR)** | Median size: 822bp (H1 cell line) |  | Two states HMM | 7.14% | Molaro et al., 2011 |
| **Low methylation region (LMR)** | Median size: 252bp (H1 cell line) | 10-50% | Three states HMM | 15.92% | Stadler et al., 2011;  Burger et al., 2013;  Xie et al., 2013 |
| **Unmethylated region (UMR)** | Median size: 532bp (H1 cell line) | <10% | Three states HMM | 0.10% | Stadler et al., 2011;  Burger et al., 2013;  Xie et al., 2013 |
|  | 1~3.5Kb | < 10% | Two states HMM | 0.21% | Jeong et al., 2014 |

**Length distribution of scUMC and other under-methylated regions**

**Table S4 Detail sentences in Window-based Method and HMM Method**

| **Reference** | **DNA methylation Regions** | **Detail Sentences**  **(Bold sentences: showing how these methods exclude/ignore scUMC)** |
| --- | --- | --- |
| Xie et al., 2013 | **DNA methylation valleys (DMV)** | To identify each DMVs in a cell type, the genome was first divided in 1kb bins and the DNA methylation level was averaged within each bin. **Then a sliding 5kb window (with 1kb step) was used to identify regions** that have an averaged methylation level less than 0.15 in a 5kb window. Continuous regions resulting from this analysis were then merged to form DMVs. |
| Jeong et al., 2014 | **DNA methylation Canyon** | The resulting NULL distribution indicates the number of CpGs required for LMR detection. With false discovery rate (FDR) at 5%, **each LMR will include at least 4 CpGs for WT HSC or at least 5 CpGs for Dnmt3a-Knockout HSC.** The UMRs are a subset of LMRs with mean methylation ratio less than 10%. UMRs less than 1kb long not used in this manuscript. UMRs greater than or equal to 3.5kb long were defined as “Canyon”. UMRs greater than or equal to 1kb but less than 3.5kb are used as control UMRs (cUMRs) to compare with Canyons to show that Canyons are very unique. |
|  | **Unmethylated region (UMR)** |  |
| Stadler et al., 2011;  Burger et al., 2013;  Xie et al., 2013 | **Low methylation region (LMR)** | Consecutive CpGs with identical state labels were combined into the same segment, and **segments with only one or two CpGs were combined with their upstream neighboring segment.** Finally, UMRs were defined as unmethylated segments, FMRs as fully methylated segments, and LMRs as low methylated segments flanked by FMRs on both sides. |
|  | **Unmethylated region (UMR)** |  |
